# Supplementary material for: Attention-deficit hyperactivity disorder symptoms and brain morphology: Examining confounding bias
Source: eLife. 2022 Nov 9;11:e78002. doi: 10.7554/eLife.78002 (PMC9708072; doi:10.7554/eLife.78002)
Supplement: Supplementary file 1. — (a) Overview of adjusted confounders for neuroimaging studies on attention-deficit/hyperactivity disorder (ADHD). Note. S = sensitivity; M = matched. (b) Descriptive statistics of the study population in the ABCD and Generation R Studies. aFor the ABCD Study, missing values were present for race/ethnicity (<0.1%), highest parental education (<0.1%), household income (8.9%), maternal age at birth (2.4%), smoking during pregnancy (<0.1%), cannabis use during pregnancy (<0.1%), and IQ (2.3%). bFor Generation R, missing values were present for ethnicity (<0.1%), maternal education (8.0%), household income (12.0%), smoking during pregnancy (11.1%), cannabis use during pregnancy (19.5%), IQ (12.4%), and aggression problems (<0.1%). [file elife-78002-supp1.docx]

**Supplementary File 1**

**Attention Deficit Hyperactivity Disorder Symptoms and Brain Morphology:**

**Examining Confounding Bias**

Lorenza Dall’Aglio^1,2^*; Hannah H. Kim^3^*; Sander Lamballais^4^*; Jeremy Labrecque^5^; Ryan L. Muetzel^1†^; Henning Tiemeier^1,3†^

^1^Department of Child and Adolescent Psychiatry, Erasmus MC University Medical Center Rotterdam-Sophia Children’s Hospital, Rotterdam, the Netherlands

^2^The Generation R Study Group, Erasmus MC University Medical Center Rotterdam, Rotterdam, the Netherlands

^3^Department of Social and Behavioral Sciences, Harvard T.H. Chan School of Public Health, Boston, USA

^4^Department of Clinical Genetics, Erasmus MC University Medical Center Rotterdam, Rotterdam, the Netherlands

^5^Department of Epidemiology, Erasmus MC University Medical Center Rotterdam, Rotterdam, the Netherlands

*Co-first author

^†^Co-last author

**Corresponding author:** Henning Tiemeier, MD Ph.D.; Department of Social and Behavioral Sciences, Harvard T.H. Chan School of Public Health, 677 Huntington Ave, Boston, 02115 MA, USA; [tiemeier@hsph.harvard.edu](mailto:tiemeier@hsph.harvard.edu)

| First Author | Year | DOI | Age | Sex | IQ | Other confounders |
| --- | --- | --- | --- | --- | --- | --- |
| Al-Amin | 2018 | <https://doi.org/10.1016/j.brainres.2018.02.007> | yes | yes | no | site, ICV |
| Vilgis | 2016 | <https://doi.org/10.1016/j.pscychresns.2016.06.008> | M | no | S | scan, ICV |
| Pineda | 2002 | [https://doi.org/10.1177/088307380201700202](https://doi.org/10.1177%2F088307380201700202) | M | M | no | weight, height, head circumference, encephalic index, SES, grade |
| Semrud-Clikeman | 2014 | [https://doi.org/10.1177/1087054714559642](https://doi.org/10.1177%2F1087054714559642) | no | no | no | total brain volume |
| Carmona | 2005 | <https://doi.org/10.1016/j.neulet.2005.07.020> | M | M | M | global activity, laterality |
| Carmona | 2009 | <https://doi.org/10.1016/j.biopsych.2009.05.013> | M | M | no | handedness |
| Saad | 2017 | <https://doi.org/10.1016/j.nicl.2017.05.016> | M | M | no |  |
| Anderson | 2014 | <https://doi.org/10.1016/j.neuroimage.2013.12.015> | M | yes | yes | site, handedness |
| Svatkova | 2016 | <https://doi.org/10.1002/hbm.23243> | S | no | S | Total brain volume |
| Ercan | 2016 | https://doi.org/10.1007/s00787-015-0731-3 | yes | yes | no |  |
| Lei | 2014 | https://doi.org/10.1038/srep06875 | M | M | no |  |
| Solanto | 2009 | <https://doi.org/10.1111/j.1552-6569.2008.00289.x> | M | M | M |  |
| Hong | 2014 | <https://doi.org/10.1016/j.biopsych.2013.12.013> | yes | yes | yes | Clinical characteristics (omission, commission errors), paternal education |
| Orinstein | 2014 | <https://doi.org/10.1016/j.pscychresns.2014.05.012> | M | M | no | Ethnicity |
| Ahn | 2015 | 10.5705/ss.2013.232w | no | no | no |  |
| Dos Santos Siqueira | 2014 | <https://doi.org/10.1155/2014/380531> | no | no | no |  |
| Fair | 2013 | <https://doi.org/10.3389/fnsys.2012.00080> | M | yes | yes | Site, movement |
| Pikusa | 2015 | <https://doi.org/10.1515/psicl-2015-0006> | S | S | no | Motion parameters |
| Sanefuji | 2016 | <https://doi.org/10.1016/j.cortex.2016.06.005> | yes | yes | yes | Head motion, site, motion |

|  | **ABCD Study**  **(N =7,722) ͣ** |  | **Generation R**  **(N = 2,531) ᵇ** |  |
| --- | --- | --- | --- | --- |
|  | **Level** | **Values** | **Level** | **Values** |
| ADHD symptoms (median, [IQR]) |  | 2.0 [0.0, 5.0] |  | 2.0 [1.0, 5.0] |
| Age at MRI assessment, years (mean ± SD) |  | 9.9 ± 0.6 |  | 10.1 ± 0.6 |
| Sex (N (%)) | Female | 3623 (46.9) | Female | 1241 (49.0) |
|  | Male | 4099 (53.1) | Male | 1290 (51.0) |
| Ethnicity (N (%)) | White | 3837 (49.7) | Western | 1842 (72.8) |
|  | Black | 1150 (14.9) | Non-western | 665 (26.3) |
|  | Hispanic | 1752 (22.7) |  |  |
|  | Asian | 182 (2.4) |  |  |
|  | Other | 792 (10.3) |  |  |
| Household income (N (%)) | < 50k | 2233 (28.9) | Low | 373 (14.7) |
|  | 50k – 100k | 2003 (25.9) | Medium | 580 (22.9) |
|  | > 100k | 2797 (36.2) | High | 1275 (50.4) |
| Highest parental education (N (%)) | No diploma | 412 (5.3) | Low | 193 (8.3) |
|  | HS diploma / GED | 778 (10.1) | Medium | 650 (27.9) |
|  | Some college | 2046 (26.5) | High | 1484 (63.8) |
|  | Bachelor | 1850 (24.0) |  |  |
|  | Post graduate | 2627 (34.0) |  |  |
|  | **Level** | **Values** | **Level** | **Values** |
| Maternal age at childbirth, years (mean ± SD) |  | 29.2 ± 6.3 |  | 31.4 ± 4.7 |
| Maternal psychopathology (median [IQR]) |  | 16 [8.0, 29.0] |  | 0.1 [0.1, 0.3] |
| Maternal smoking during pregnancy (N (%)) | No | 6485 (84.0) | No | 1784 (70.5) |
|  | Yes | 1034 (13.4) | Yes | 466 (18.4) |
|  | Don’t know | 201 (2.6) |  |  |
| Maternal cannabis during pregnancy (N (%)) | No | 7017 (90.9) | No | 1991 (78.6) |
|  | Yes | 470 (6.1) | Yes | 46 (1.8) |
|  | Don’t know | 233 (3.0) |  |  |
| IQ (mean ± SD) |  | 9.9 ± 3.0 |  | 103.9 ± 14.7 |
