# Supplementary material for: Attention-deficit hyperactivity disorder symptoms and brain morphology: Examining confounding bias
Source: eLife. 2022 Nov 9;11:e78002. doi: 10.7554/eLife.78002 (PMC9708072; doi:10.7554/eLife.78002)
Supplement: Reporting standard 2. [file elife-78002-repstand2.docx]

**STROBE Flowchart**

Flowcharts of participant inclusion and exclusion for ABCD (panel A) and Generation R (panel B).


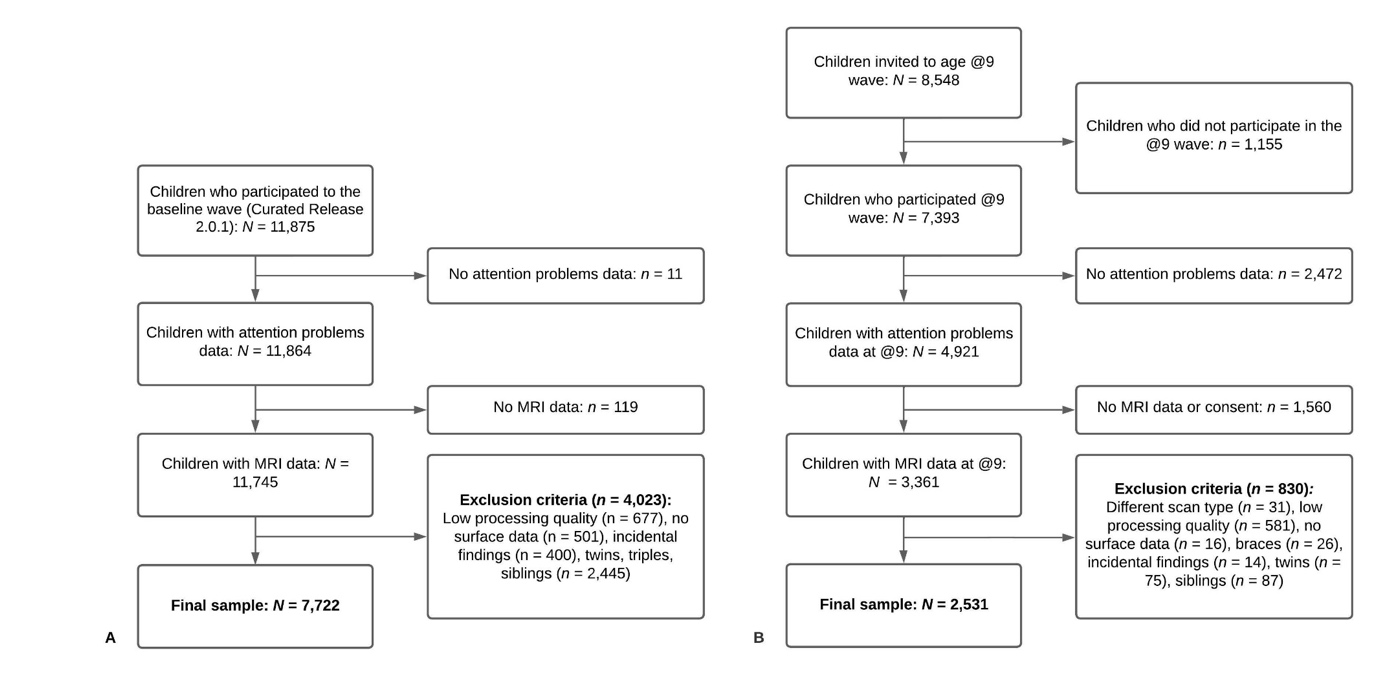


*Note.* **A.** In the ABCD Study, of the 11,875 participants enrolled in the study, 7,722 met our inclusion and exclusion criteria. **B.** In the Generation R Study, of the 8,548 participants invited to the age 9-10 assessment, 2,531 met our inclusion and exclusion criteria.
