# Supplementary material for: Attention-deficit hyperactivity disorder symptoms and brain morphology: Examining confounding bias
Source: eLife. 2022 Nov 9;11:e78002. doi: 10.7554/eLife.78002 (PMC9708072; doi:10.7554/eLife.78002)
Supplement: Transparent reporting form [file elife-78002-transrepform1.pdf]

eLIFE
1st Floor
24 Hills Road
Cambridge CB2 1JP, UK
P 01223 855340
W elifesciences.org
T @elife

eLife's transparent reporting form

We encourage authors to provide detailed information *within their submission* to facilitate the interpretation and replication of experiments. Authors can upload supporting documentation to indicate the use of appropriate reporting guidelines for health-related research (see EQUATOR Network), life science research (see the BioSharing Information Resource), or the ARRIVE guidelines for reporting work involving animal research. Where applicable, authors should refer to any relevant reporting standards documents in this form.

If you have any questions, please consult our Journal Policies and/or contact us: editorial@elifesciences.org.

Sample-size estimation

- You should state whether an appropriate sample size was computed when the study was being designed
- You should state the statistical method of sample size computation and any required assumptions
- If no explicit power analysis was used, you should describe how you decided what sample (replicate) size (number) to use

Please outline where this information can be found within the submission (e.g., sections or figure legends), or explain why this information doesn't apply to your submission:

While no power analysis was conducted, we used the largest in-site and multi-site studies of neurodevelopment. The sample sizes used reflected the available data on MRI and behavioural measures for each cohort. Data was independently collected by the ABCD and Generation R Study teams. This manuscript constitutes a secondary data analysis.

Replicates

- You should report how often each experiment was performed
- You should include a definition of biological versus technical replication
- The data obtained should be provided and sufficient information should be provided to indicate the number of independent biological and/or technical replicates
- If you encountered any outliers, you should describe how these were handled
- Criteria for exclusion/inclusion of data should be clearly stated
- High-throughput sequence data should be uploaded before submission, with a private link for reviewers provided (these are available from both GEO and ArrayExpress)

Please outline where this information can be found within the submission (e.g., sections or figure legends), or explain why this information doesn't apply to your submission:

Biological replication is not applicable.

We ensured we could reproduce the results by rerunning the analyses 3 times. Additionally, we used two independent samples to evaluate replicability. Outliers were not identified nor removed in this study, as the ABCD and Generation R Study teams independently check for any outlier or invalid data. Criteria for inclusion and exclusion are specified in the Methods section and shown in a flowchart in Supplementary Figure 9.

The high-throughput sequence data does not apply to this submission.

eLife Sciences Publications, Ltd is a limited liability non-profit non-stock corporation incorporated in the State of Delaware, USA, with company number 5030732, and is registered in the UK with company number FC030576 and branch number BR015634 at the address 1st Floor, 24 Hills Road, Cambridge CB2 1JP | August 2014

Statistical reporting

- Statistical analysis methods should be described and justified
- Raw data should be presented in figures whenever informative to do so (typically when N per group is less than 10)
- For each experiment, you should identify the statistical tests used, exact values of N, definitions of center, methods of multiple test correction, and dispersion and precision measures (e.g., mean, median, SD, SEM, confidence intervals; and, for the major substantive results, a measure of effect size (e.g., Pearson's r, Cohen's d)
- Report exact p-values wherever possible alongside the summary statistics and 95% confidence intervals. These should be reported for all key questions and not only when the p-value is less than 0.05.

Please outline where this information can be found within the submission (e.g., sections or figure legends), or explain why this information doesn't apply to your submission:

This information can be found in the Results section where the clusters for associations are shown. Effect sizes are also depicted across the brain and across regions in the Results section and in the Supplementary File.

(For large datasets, or papers with a very large number of statistical tests, you may upload a single table file with tests, Ns, etc., with reference to sections in the manuscript.)

Group allocation

- Indicate how samples were allocated into experimental groups (in the case of clinical studies, please specify allocation to treatment method); if randomization was used, please also state if restricted randomization was applied
- Indicate if masking was used during group allocation, data collection and/or data analysis

Please outline where this information can be found within the submission (e.g., sections or figure legends), or explain why this information doesn't apply to your submission:

NA

Additional data files ("source data")

- We encourage you to upload relevant additional data files, such as numerical data that are represented as a graph in a figure, or as a summary table
- Where provided, these should be in the most useful format, and they can be uploaded as "Source data" files linked to a main figure or table
- Include model definition files including the full list of parameters used
- Include code used for data analysis (e.g., R, MatLab)
- Avoid stating that data files are "available upon request"

Please indicate the figures or tables for which source data files have been provided:

Several figures have been provided in-text and in the Supplementary File to represent the data summary statistics.

All datasets for this article are not automatically publicly available due to legal and informed consent restrictions. Reasonable requests to access the datasets should be directed to the Director of the Generation R Study, Vincent Jaddoe (generationr@erasmusmc.nl), in accordance with the local, national, and European Union regulations. Data for The ABCD Study is already open and available in the NIMH Data Archive (NDA) (nda.nih.gov) to eligible researchers within NIH-verified institutions. Data can be accessed following a data request to the NIH data access committee (https://nda.nih.gov/), which should include information on the planned topic of study. The request is valid for one year. Data use should be in line with the NDA Data Use Certification. The code used for this study is publicly available at https://github.com/LorenzaDA/ADHD_brainmorphology_confounding
